# Morphological and molecular characterization of *Epidorylaimus procerus* sp. n. (Dorylaimida: Qudsianematidae) from Vietnam

**DOI:** 10.21307/jofnem-2020-112

**Published:** 2020-11-06

**Authors:** Thi Anh Duong Nguyen, Reyes Peña-Santiago

**Affiliations:** 1 Institute of Ecology and Biological Resources, Vietnam Academy of Science and Technology, 18 Hoang Quoc Viet, Cau Giay, Hanoi, Vietnam; 2 Departamento de Biología Animal, Biología Vegetal y Ecología, Universidad de Jaén, Campus ‘Las Lagunillas’ s/n, Edificio B3, 23071, Jaén, Spain; 3 Graduate University of Science and Technology, Vietnam Academy of Science and Technology, 18 Hoang Quoc Viet, Cau Giay, Hanoi, Vietnam

**Keywords:** D2-D3 28S-rRNA, Description, Morphology, Morphometry, Nematode, New species, Taxonomy, Vietnam

## Abstract

*Epidorylaimus procerus* sp. n., collected from a natural habitat in Vietnam, is described and illustrated. It is distinguishable by its 2.16 to 2.46-mm-long body, lip region offset by depression and 15 to 17-µm broad, odontostyle 32 to 35-µm long, neck 415 to 461-µm long, pharyngeal expansion occupying 47 to 52% of the total neck length, uterus 76 to 130-µm long or 1.0 to 1.5-body diameters, vulva transverse (*V* = 40-43), caudal region conical elongate (157-186 µm, *c* =12.1-14.4, *c′* = 4.4-5.5) with blister-like bodies, and hyaline portion occupying one-fourth its length, and males absent. Molecular analysis shows a close relationship of the new species and *E. lugdunensis*, supporting monophyly of the genus *Epidorylaimus*.

The genus *Epidorylaimus* Andrássy, 1986 is a widespread taxon in Dorylaimida. Originally proposed to accommodate 12 species previously included in *Eudorylaimus* Andrássy, 1959 its taxonomy was later updated by several authors (Andrássy, 1992; Jairajpuri and Ahmad, 1992; Vinciguerra, 2006; Andrássy, 2009). More recently, Ahmad et al. (2016) presented an emended diagnosis, a list of 15 valid species with a key to their identification, and a compendium of their more relevant morphometrics.

In spite of the nearly cosmopolitan distribution of the genus, none of its species was hitherto recorded in Vietnam. However, a nematological survey conducted to study nematode fauna associated with natural habitats in the country located an interesting *Epidorylaimus* species. Its study revealed that it belonged to an unknown species that is hereunder described.

## Materials and methods

### Nematode extraction and processing

Soil samples were collected in Cao Bang Province, a natural area of Northern Vietnam, and temporarily stored in plastic bags for transport to the laboratory. Nematode extraction was done following the methods of Baermann (1917) and Flegg (1967). Specimens were relaxed and killed with heat, fixed in 4% formaldehyde, processed to anhydrous glycerin according to Siddiqi’s (1964) technique, and mounted on permanent glass slides for handling and observation with light microscopy.

### Light microscopy (LM)

Specimens were measured, drawn, and identified with a Nikon Eclipse 80i light microscope equipped with differential interference contrast (DIC) optics and a drawing tube (*camera lucida*). Morphometrics included Demanian ratios and other measurements. Microphotographs were taken with the same microscope provided with a Nikon Digital Sight DS-U1 camera. Raw photographs were edited using Adobe^®^ Photoshop^®^ CS.

### DNA extraction, PCR, and sequencing

DNA was extracted from single individuals using the proteinase K and Worm Lysis Buffer protocol (William et al., 1992). Each nematode was transferred to a 0.5-ml Eppendorf tube containing 18 µl of Worm Lysis Buffer (WLB) (50 mM KCL, 10 mM Tris, pH 8.3, 2.5 mM MgCl_2_, 0.45% NP 40, and 0.45% Tween 20) and 2 µl of proteinase K (600 µg ml^−1^) (Thermo Scientific). The tubes were incubated at 65°C (1 hr) and then at 95°C (15 min). PCR was performed in a 30-µl final volume containing 24.9 µl of sterile water, 0.6 µl of each PCR primer, 0.6 µl of dNTP, 0.3 µl of Taq-polymerase, 3 µl of Buffer 10 x Thermo Scientific Green, and 1 µl of DNA-extracted solution. The PCR amplification profile consisted of 4 min at 94°C, 35 cycles of 1 min at 94°C, 1.5 min at 55°C, and 2 min at 72°C, followed by a final step of 10 min at 72°C. The primers used for amplification were D2A (5´-ACAAGTACCGTGAGGGAAAGTTA-3´) and D3B (5´-TCCTCGGAAGGAACCAGCTACTA-3´) for amplification of the D2-D3 region of 28S (Subbotin et al., 2006).

PCR products were purified with the GeneJET PCR Purification Kit (#K0701, Thermo Scientific, USA), following the manufacturer’s manual. The sequencing reaction was performed with 15 ng of purified template, 4 µL of BigDye Terminator v3.1 Ready Reaction Mix, 2 µL of 5X Sequencing Buffer, and 3.2 pmol of forward/reverse primers for a total of 10-µL volume. The mixture was heated for 10 sec at 96°C, then 5 sec at 55°C, repeated for 32 cycles followed by 4 min at 60°C. The sequencing was performed on 3500 x L Genetic Analyzers (Applied Biosystems, Foster City, California) at the National Key Laboratory of Gene Technology (IBT – VAST, Hanoi). The sequences obtained were submitted to the GenBank database under accession numbers MT612084 and MT612088.

### Phylogenetic analysis

The obtained sequences were aligned with 35 other D2-D3 expansion segments of 28S rDNA gene sequences available in GenBank, using SeaView with the muscle algorithm followed by manual refining (V4.5.3, Gouy et al., 2010). Outgroup taxa were chosen according to previously published data (Holterman et al., 2008; Álvarez-Ortega et al., 2013). The sequence dataset was analyzed with Bayesian inference (BI) and maximum likelihood (ML) using MrBayes 3.1.2 (Huelsenbeck and Ronquist, 2001; Ronquist and Huelsenbeck, 2003). We chose the commonly used model GTR + I + G that then was used to calculate phylogenetic trees in PhyML 3.1 with 100 replicates and MrBayes with a burn-in of 25% and the final split frequencies of less than 0.01 (settings: mcmc ngen = 1,000,000; sample freq = 500; print freq = 500; diagn freq = 5,000; Ronquist and Huelsenbeck, 2003; Altekar et al., 2004). Both trees where then combined into one reconstructed phylogeny in Adobe Illustrator^©^ (Creative Suite 6).

## Results

*Epidorylaimus procerus* sp. n.

(Figs. 1 and 2)

**Figure 1:**
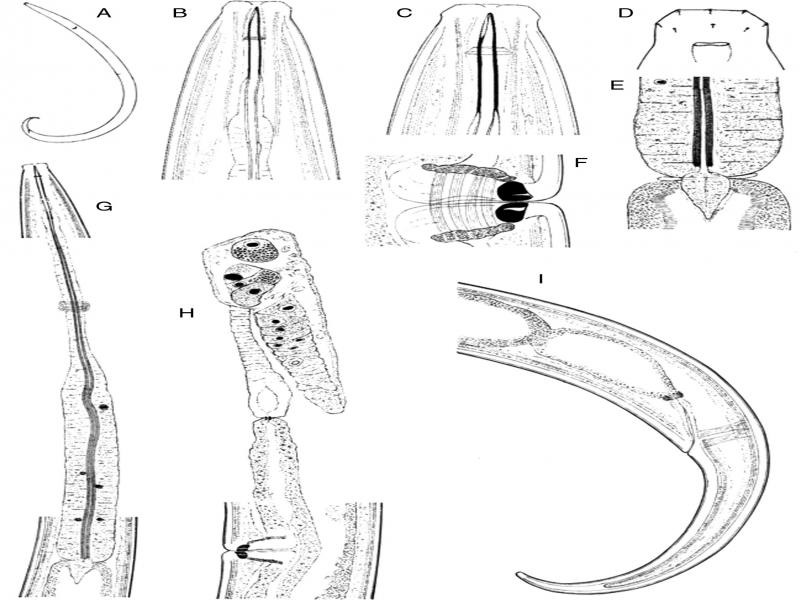
*Epidorylaimus procerus* sp. n. (female). A: Entire. B, C: Anterior region in lateral median view. D: Lip region in lateral surface view. E: Pharyngo-intestinal junction. F: Vagina. G: Neck region. H: Anterior genital branch. I: Posterior body region [scale bars: A = 500 µm; B, C = 10 µm; D, F = 5 µm; E = 20 µm; G-I = 50 µm].

**Figure 2:**
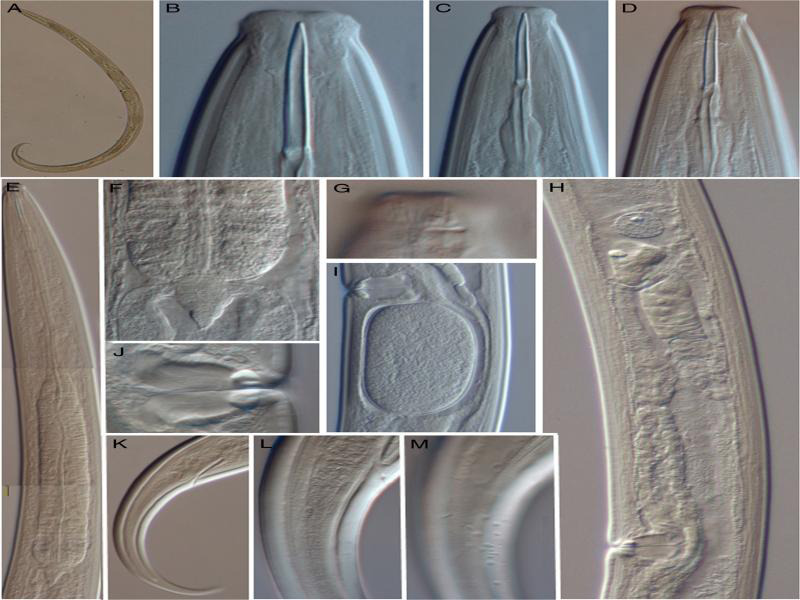
*Epidorylaimus procerus* sp. n. (female, LM). A: Entire. B-D: Anterior region in lateral median view. E: Neck region. F: Pharyngo-intestinal junction. G: Lip region in lateral surface view. H: Anterior genital branch. I: Uterine egg. J: Vagina. K: Caudal region. L: Anterior portion of the caudal region in median view. M: Same in submedian view, showing the blister-like elements mainly subventrally and ventrally [scale bars: A = 500 µm; B-D = 10 µm; E, H, K = 50; F, I, L, M = 20 µm; G, J = 5 µm].

### Material examined

In total, there were 15 females from one location in excellent state of preservation.

Morphometricssee Table 1.

**Table 1.** Morphometrics of *Epidorylaimus procerus* sp. n. from Vietnam.

	Holotype	Paratypes
	♀	15♀♀
Character		
*L*	2.37	2.28 ± 0.09 (2.16-2.46)
*a*	30.8	31.4 ± 1.2 (28.8-32.9)
*b*	5.5	5.2 ± 0.2 (4.9-5.6)
*c*	13.0	13.3 ± 0.7 (12.1-14.4)
*V*	41.5	41.2 ± 0.8 (39.8-42.6)
*c′*	5.4	4.9 ± 0.4 (4.4-5.5)
Lip-region diameter	17	15.9 ± 0.6 (15-17)
Odontostyle length	32	33.4 ± 1.2 (32-35)
Odontophore length	32	30.2 ± 1.5 (28-33)
Neck length	432	435 ± 18 (415-461)
Pharyngeal expansion length	204	212 ± 13 (194-238)
Body diam. at neck base	61	65.0 ± 4.0 (61-73)
Mid-body	77	72.6 ± 3.4 (68-79)
Anus/cloaca	36	35.2 ± 1.3 (32-36)
Distance vulva – anterior end	982	938 ± 43 (885-1012)
Prerectum length	91	91.8 ± 18.4 (70-123)
Rectum length	42	42.5 ± 4.6 (35-49)
Tail length	182	171 ± 9.2 (157-186)

**Note:** Measurements are in μm, except *L* in mm, and paratype measurements are average ± sd (range).

### Description

#### Female

There were moderately slender (*a* = 29-33) nematodes of medium size, 2.16-2.46-mm long. Their body was cylindrical, tapering anteriorly, extended posteriorly as an elongate, conical, and had a curved tail. Upon fixation, the habitus slightly curved ventrad, C- to hook-shaped. The cuticle was two-layered, 2 to 3-µm thick at the anterior region, 3.5 to 4 µm in mid-body, and 6.5 to 8.5 µm on the tail; the outer layer was thin and smooth, with constant thickness throughout the body; the inner layer was much thicker than the outer layer, which was especially conspicuous at the tail. The lateral chord was 15 to 19-µm broad, occupying 21 to 27% of mid-body diameter. Body pores were obscure.

The lip region was truncate, offset by a distinct constriction, 1.9 to 2.3 times broader than high and one-fifth to one-fourth (21-26%) of body diameter at the neck base; the lips were amalgamated, with visibly protruding labial and cephalic papillae. Amphid fovea appeared to be cup-like, its aperture measured 4 to 5.5 µm, occupying up to one-third (28-33%) of lip-region diameter. Cheilostom was a truncate cone, without any particular differentiation. Odontostyle was relatively long and slender, 10.7 to 11.5 times longer than wide, length about twice (1.9-2.1 times) the diameter of the lip-region diameter, and about 1.38 to 1.51% of the total body length; the aperture measured 6.5 to 8.0-µm long, occupying up to one-fourth (20-25%) of odontostyle length. Odontophore was rod-like, nearly equal (0.8-1.0 times) in length to odontostyle. The pharynx was entirely and conspicuously muscular, with its slender portion enlarging gradually, and the basal expansion being 5.4 to 6.5 times as long as wide, 3.0 to 3.6 times longer than body diameter at the neck base, and occupying *ca* one-half (47-52%) of neck length; gland nuclei are located as follows: DO = 56 to 63, DN = 59 to 65, S_1_N_1_ = 77 to 80, S_1_N_2_ = 78 to 83, and S_2_N = 89 to 90. The nerve ring was situated at 152 to 165 µm or 34 to 37% of the total neck length from the anterior end. The pharyngo-intestinal junction consisted of a well-developed, conical cardia measuring 19 to 29 × 11 to 15 µm, enveloped by the intestinal wall. The intestine lacked any relevant differentiation. The genital system was didelphic-amphidelphic, with both branches equally developed, anterior 212 to 278 µm or 10 to 13%, posterior 206 to 324 µm or 9 to 14% of the total body length. The ovaries were reflexed, the anterior measured 122 to 165 µm, and the posterior 140 to 229-µm long, often reaching and surpassing the oviduct-uterus junction, with oocytes arranged first in two or more rows and then in a single row. The oviduct was 91 to 132-µm long or 1.3 to 1.8-body-diameter long, consisting of a distal, slender section made of prismatic cells and a poorly developed proximal *pars dilatata* with visible lumen inside. A narrowing surrounded by a muscular ring (sphincter) separates the oviduct and the uterus. The uterus is a simple, tube-like structure measuring 76 to 130 µm or 1.0 to 1.5-body-diameter long. The vagina extended inward from 28 to 34 µm, occupying 39 to 46% of body diameter: *pars proximalis* 18-25 × 14-21 µm, and was surrounded by moderately developed circular musculature; *pars refringens* consisted of (in lateral view) two trapezoidal sclerotized pieces, 3.5-6 × 6-7 µm, with a combined width of 12.5 to 14.5 µm; *pars distalis* measured 4.5 to 6.5-µm long. Vulva was a transverse slit. Prerectum 2.0-3.4x, rectum 1.0-1.4x times the anal body diameter long. The tail was conically elongated, gradually tapering to a finely rounded tip, and strongly curved ventrad, with abundant saccate- or blister-like bodies inside the cuticle at its anterior third; two pairs of caudal pores, one subventral, another subdorsal, at about one anal-body diameter behind the level of the anus; the inner core could not reach the tail tip; therefore, a hyaline portion exists measuring 36 to 48 -µm long and occupying *ca* one-fourth (22-28%) of the total tail length.

#### Male

Unknown.

### Diagnosis

The new species is distinguished from other *Epidorylaimus* spp. by a combination of the following character states and morphometrics: body 2.16 to 2.46-mm long, lip region offset by constriction, width 15 to 17-µm broad, odontostyle 32 to 35-µm long with aperture occupying 20 to 25% of its length, neck 415 to 461-µm long, pharyngeal expansion 194 to 238-µm long (47-52% of the total neck length); the female genital system was described as follows: didelphic-amphidelphic, uterus simple and 76 to 130-µm long (1.0-1.5 body diameters), and vulva transverse (*V* = 40-43); the caudal region was conically elongated (157-186 µm, *c* = 12.1-14.4, *c′* = 4.4-5.5) with blister-like bodies and hyaline portion occupying one-fourth its length.

### Relationships

*Epidorylaimus procerus* sp. n. resembles *E. mellenbachensis* (Alther, 1974) Andrássy, 1986 and *E. rivalis* Gagarin, 1991., and can be easily distinguished from other species of the genus (see key and table compendium by Ahmad et al., 2016) in having odontostyle longer than 25 µm. It differs from the German freshwater species *E. mellenbachensis* in its larger general size (body length 2.16-2.46 vs 1.70-2.00), less slender body (*a* = 29-33 vs 42-50), longer odontostyle (32-35 vs 28-29 µm; 1.9-2.1 vs 1.25 times lip-region diameter), much more anterior vulva (*V* = 39-42 vs *V* = 50-55), and relatively longer tail (*c* = 12-14 vs *c* = 19-38; *c′* = 4.4-5.5 vs *c′* = 3-4). From the Russian species *E. rivalis*, it differs in being longer (2.16-2.46 vs 1.46-2.29 mm, *n* = 15), the lip region is less differentiated (weak vs deep constriction), and it has a longer odontostyle (32-35 vs 28-30 µm). Males of *E. rivalis* are as abundant as females, whereas they are unknown in *E. procerus* n. sp. and thus likely to be rare or absent.

**Figure 3:**
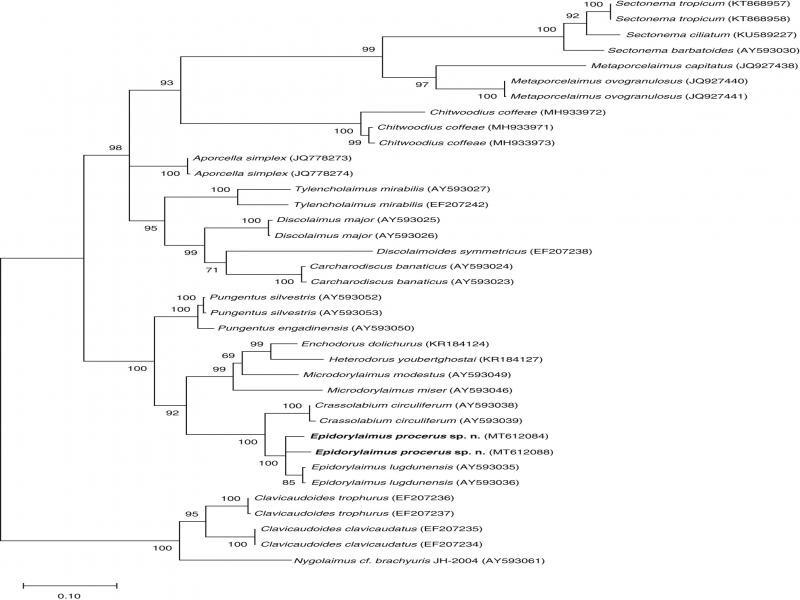
Combined Bayesian inferred and maximum likelihood tree of *Epidorylaimus procerus* sp. n. based on sequences of the 28S rDNA region.

Evolutionary relationships, as derived from the analysis of D2-D3 28S-rRNA gene sequences, are presented in a molecular tree (Fig. 3). The new species forms part of a highly supported (99.9%) clade with *E. lugdunensis* de Man, 1880, the type species of the genus, supporting monophyly of *Epidorylaimus*. Both species appear closely related (100% support) to *Crassolabium circuliferum* Loof, 1961, a member of the family Dorylaimidae, and form part of a highly supported larger clade that includes members of the families Nordiidae and Qudsianematidae.

### Type locality and habitat

Vietnam, Cao Bang Province, Cao Bang Natural Reserve (GPS coordinates: 22° 34′07″ N, 105° 52′34″ E), in a tropical evergreen forest soil with *Dipterocarpus* sp. and *Cinnamomum* sp. as dominant plant species.

### Type material

Female holotype and nine female paratypes were deposited with nematode collection of the University of Jaén, Spain. Five female paratypes were deposited with the nematode collection of the Institute of Ecology and Biological Resources (IEBR), Hanoi, Vietnam.

### Etymology

The specific epithet is the Latin term *procerus* = slim or svelte, and refers to the slender figure of these nematodes.
